# PMA-Zeolite: Chemistry and Diverse Medical Applications

**DOI:** 10.3390/jfb15100296

**Published:** 2024-10-04

**Authors:** Aleksandar Bulog, Kresimir Pavelic, Ivana Šutić, Sandra Kraljevic Pavelic

**Affiliations:** 1 Teaching Institute for Public Health of Primorsko-Goranska County, Krešimirova ulica 52, 51000 Rijeka, Croatia; aleksandar.bulog@gmail.com (A.B.); ivana.sutic@medri.uniri.hr (I.Š.); 2 Faculty of Medicine, University of Rijeka, Braće Branchetta 20, 51000 Rijeka, Croatia; 3 Faculty of Medicine, Juraj Dobrila University of Pula, Zagrebačka 30, 52100 Pula, Croatia; pavelic@unipu.hr; 4 International Academy of Sciences and Arts in Bosnia and Herzegovina (IANUBIH), Radnička Cesta 34, 71000 Sarajevo, Bosnia and Herzegovina; 5 Faculty of Health Studies, University of Rijeka, Ulica Viktora Cara Emina 5, 51000 Rijeka, Croatia

**Keywords:** clinoptilolite, PMA-zeolite in medicine, orthosilic acid, systemic biological effects, applications in clinics

## Abstract

Numerous scientific studies have been conducted in recent decades with the aim to study targeted application of zeolites in various industries, ecology, agronomy and medicine. The biggest advances, however, have been documented in medical and veterinary research of the natural zeolite, clinoptilolite. Although the exact biological mechanisms of action of the zeolite clinoptilolite are not completely elucidated, obtained results point to its antioxidative, immunomodulatory and detoxifying effects, the latter partially based on release of soluble and bioavailable silica forms from the surface material. The studied zeolite clinoptilolite materials have different geographical origins which confer to the physicochemical differences in the material. In addition, the production process of the material for oral applications differs between different producers which also accounts for different properties of the surface upon mechanical activation. Recently, a well-characterized zeolite clinoptilolite material, namely the PMA-zeolite, has been tested in different clinical applications and has shown potential as supportive therapy in inflammatory conditions, osteoporosis as well as during tumor chemotherapy. We accordingly present a comprehensive review of the PMA-zeolite effects in the clinical applications and discuss its probable mechanisms of effect in vivo.

## 1. Introduction

Long-term scientific research results provide a solid amount of evidence on the antioxidant and immunomodulating effects of certain types of zeolites, primarily clinoptilolite, in medical and veterinary applications. In such applications, the zeolite clinoptilolite is mainly used in the form of oral supplements [1] or as an ingredient within various products for oral hygiene [2] or skin applications [3]. The zeolite clinoptilolite mineral has unique properties that are growingly exploited in medical applications. Many natural zeolites show interesting biological effects as well, such as, for example, bentonite [4,5] or chabazite [6]. Still, clinoptilolite zeolite remains the most studied one. The structure of the zeolite clinoptilolite material (for details please refer to [7,8]), underlies its physical–chemical characteristics. This inorganic material can accordingly be distinguished clearly from other medical substances that have an organic basis and thus a completely different effect in the organism. Indeed, small molecules and organic molecules are studied and developed in the context of their corresponding implications to the metabolism and biochemical pathways in living cells. This is not the case for zeolite materials.

In this review, accordingly, we present scientific evidence of positive biological effects of one defined natural zeolite clinoptilolite material, the PMA-zeolite, gathered through a number of clinical studies. Previously, concerns were raised on the usage of the clinoptilolite-based materials in humans due to a lack of scientific studies on the material safety and effects in vivo. The herein described and discussed studies shed light on the mechanisms of action and effects in vivo of the PMA-zeolite material that might be readily used by clinicians and open new avenues for additional clinical research in the future. As the effects of this material are closely correlated with its chemical structure and physical–chemical properties, we explain briefly the clinoptilolite materials’ structural properties, i.e., crystal structure, porosity and ion-exchange properties below.

The aluminosilicate microporous structure of clinoptilolite is based on the organization of the aluminum oxide and silicate tetrahedra that are connected by oxygen atoms. Such structure gives rise to a hydrated crystal lattice with pores and channels with a negative surface charge. The negative charge is compensated by positively charged ions of alkali and alkaline earth metals, which are also hydrated and bind to the structure through electrostatic forces. The general chemical formula of clinoptilolite is (K, Na, Ca)6[(Si, Al)36O72] nH2O (n = 20−24) [9]. Such structure, documented by the International Zeolite Association (http://www.iza-online.org/natural/Datasheets/Clinoptilolite/clinoptilolite.htm, accessed on 20 August 2024.) underlies the main clinoptilolite properties including adsorption, ion-exchange, catalytic properties and molecular sieve properties. Accordingly, the majority of studies conducted with the zeolite clinoptilolite in industrial applications include the ion-exchange of toxic cations, reduction in water hardness and water purification, absorbent applications for various gasses such as ammonia, hydrogen sulfide, nitrogen oxides, sulfur oxides and carbon dioxide or biocatalysts applications [10,11,12,13,14]. Recently, synthetic clinoptilolite materials have also gained interest. In particular, synthesis, modification of the material surface and broader applications of synthetic clinoptilolite materials have been performed and were reviewed by Ambrozova et al. [15] (Table 1). However, this field needs further research and analysis of the toxicity, biodistribution and systemic biological effects as the new materials may have unknown effects or unknown fate in the human body.

The natural zeolite clinoptilolite raw material occurs as a rock. Its content is, accordingly, highly dependent on the rock’s petrography and forming conditions. In addition, the mineralization process of zeolite occurs often with other components. Some of these components are, for example, clay minerals and feldspar or zircon and chlorite minerals [9]. The mined raw zeolite clinoptilolite material is also subject to the control of physical–chemical properties (i.e., porosity, alkali metal content) and surface properties that arise from mechanical activation via different milling technologies. This mechanical process is necessary in mechanical activation of the material for oral applications, but it also impacts the physical and chemical properties of the zeolite material [7,25]. Accordingly, each clinoptilolite product will have distinct physical and chemical properties due to specific chemical and physical features of the finally prepared material [26]. Surface changes in the clinoptilolite material might underlie the biological effects as well, which makes it plausible to avoid generalizations of biological effects for similar materials and products and to study well-characterized materials in defined medical applications. Currently, various clinoptilolite-based products are used in medicine. These materials contain different percentages of clinoptilolite and different mineral compositions. Also, natural tuffs containing clinoptilolite contain small quantities of other trace elements, including diverse quantities of various cations in the material pores. Some of the biologically important alkaline ions contained in the crystal lattice, Na^+^, Ca^2+^, Mg^2+^ and K^+^, can be easily released during the ion-exchange process [8]. This process, however, strongly depends on pore and channels size, extra-framework exchange sites and the possibility of access to exchangeable cations from the outside.

One of the well-studied and characterized materials is the zeolite clinoptilolite, registered in EU as a medical device under the name PMA-Zeolite as per European Union directive 93/42/EEC. PMA-Zeolite has been researched over more than a decade for its biomedical applications and potential mechanism of action in vivo. Previously published results compared the PMA-Zeolite material with different clinoptilolite materials and the results showed that each tested clinoptilolite material differed in physicochemical properties. These properties are related to the method of production. The studies also confirmed the safety of the biological use of PMA-Zeolite since no leaching into the blood or organs of the tested animals was observed for aluminum [7]. Accordingly, we herein present obtained data for this particular product from the published literature and discuss possible mechanisms of action underlying the observed in vivo effects (Table 2).

Importantly, zeolite clinoptilolite materials prepared via the micronization of the raw material have been shown not to enter into the body’s bloodstream [27]. Direct effects are thus confined to the gastrointestinal tract events after zeolite clinoptilolite ingestion. As the conditions, and especially the pH value and digestive enzymes, differ substantially in each compartment, we hypothesize the changes in the material that give rise to systemic effects as follows. It is indeed well-known that HCl is the most commonly used acid for activation (increase) of the clinoptilolite surface area. For example, treatment with 2M HCl may increase the surface area from 13 m^2^/g to 78 m^2^/g [28]. In the oral cavity, the pH is near neutral and the release of cations, particularly Na^+^ and Ca^2+^, through the ion-exchange process into the surroundings may result in the removal of eventual harmful substances and increased pH values in the oral cavity. In the acidic pH of the stomach, the negative zeolite surface adsorbs H^+^ cations and pH increase consequently. When the zeolite concentration is high, processes such as, for example, complexion or hydrolysis of cations may occur. These chemical reactions underlie the antacid effect [29]. In the small intestine, the pH is higher than in the stomach and promotes selective ion-exchange and adsorption. The surface of clinoptilolite changes in the acidic pH and aluminum and silicon species may be released as described previously. Particularly, the soluble silica forms underlie the clinoptilolite detoxification properties toward aluminum [7]. It is worth noting that selective ion-exchange properties of clinoptilolite materials are particularly interesting in the removal of radioactive cations Cs^+^ and Sr^2+^. The main factor that influences the adsorption of these cations on zeolites is the content of clinoptilolite in the material and a smaller particle size upon micronization [30].

## 2. PMA Ion-Exchange and Adsorption Properties

**Figure 1:**
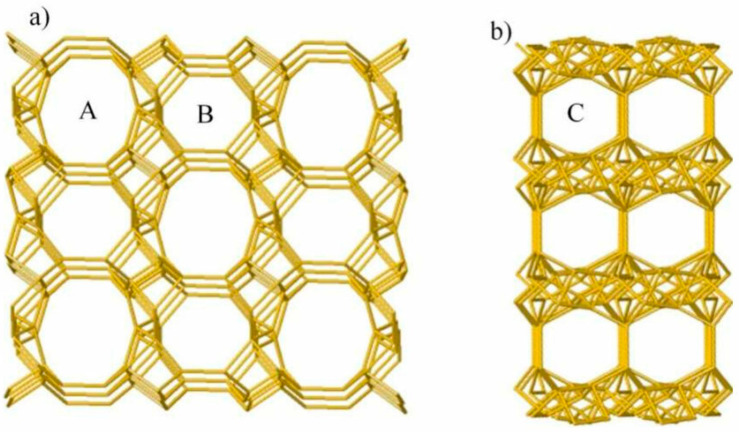
Three-dimensional structure of clinoptilolite, (**a**) crystallographic direction [001] and (**b**) crystallographic direction [100]. Three types of channels are visible: The parallel channels A (0.72 nm × 0.44 nm) and B (0.41 nm × 0.44 nm) and C (0.40 nm × 0.55 nm) that intersects channels A and B. Courtesy from Basic Treatment in Natural Clinoptilolite for Improvement of Physicochemical Properties. 2018, Minerals 8(12):595 https://doi.org/10.3390/min8120595 (accessed on 28 August 2024), available via license: Creative Commons Attribution 4.0 International [32].

The ion-exchange property of zeolites, including the zeolite clinoptilolite, is characteristic of other crystalline silicate minerals as well, i.e., clays and feldspars. The structural feature underlying this property is the isomorphic substitution of Si atoms in the structure by other atoms; in the case of zeolite clinoptilolite, it is with the Al ions. This substitution of Si^4+^ cations with Al^3+^ cations gives rise to excess of negative charge of the framework. This charge disbalance is compensated by cations, and electroneutrality of the zeolite structure is accordingly secured. The cations reside in the extra-framework sites, pores and channels and are thus easily exchanged with the surrounding environment with other available cations. The zeolite clinoptilolite material has a micropore structure consisting of channels A (0.72 × 0.44 nm), B (0.55 × 0.40 nm) and C (0.41 × 0.40 nm). These channels are formed by alternating 10- and 8-membered ring frameworks [31] (Figure 1). This applies to the PMA-Zeolite as well.

The ion-exchange property of the zeolite clinoptilolite depends strongly on the initial composition of the material, and each particular application in this field requires previous studies of the total ion-exchange capacity and effects in certain set-ups (for detailed interpretation of ion-exchange properties in correlation with material properties, please see: [32,33]). In particular, assessment of the in vivo effects in humans may be extremely challenging as the intestinal environment is complex, comprising compartments with different pH values, digestive juices with various enzymes, presence of bile acids and minerals as well as an extremely interpersonal variable microbiota content. The intestinal environment thus continuously interacts with the zeolite clinoptilolite surface during its passage through the intestine until being extracted through feces out of the body. This is why the gold standard method—inductively coupled plasma mass spectrometry (ICP-MS)—may be used for the measurement of metal contaminants and mineral levels in the blood, urine and feces during clinoptilolite intake. PMA-Zeolite was accordingly tested for such effects in humans receiving PMA-Zeolite within three clinical trials with different supplementation regimens. Through using ICP-MS, levels of metal contaminants and minerals were measured in the blood and urine of tested subjects. The regimen of PMA-Zeolite intake comprised a short-term supplementation in healthy volunteers (28 days), a medium-term supplementation in Morbus Crohn patients (12 weeks), and a long-term supplementation in osteoporosis patients (4 years) [34]. The levels of lead (Pb) were found to be elevated in the short-term trials and long-term supplementation upon PMA-Zeolite supplementation. It is important to note, however, that these levels decreased in the long-term supplementation regime. This is probably indicative of initiation of the Pb clearance from the body and its mobilization from body compartments according to the known Pb pharmacodynamics. This hypothesis is corroborated by the fact that no leakage of aluminum (Al) or lead (Pb) from the material was observed in the blood 1 h after PMA-Zeolite ingestion. In the regime of long-term PMA-Zeolite supplementation, two contaminants’ levels were substantially decreased, namely nickel (Ni) and aluminum (Al). Interestingly, a decrease in arsenic (As) levels was observed only in the medium-term supplementation. From a clinical point of view, two physiologically relevant minerals were decreased in patients with osteoporosis in the long-term supplementation, namely sodium (Na) and calcium (Ca). This observation was discussed by authors as a possible consequence of the bone remodeling processes [34]. Two animal studies confirmed PMA-Zeolite effects on the removal of aluminum in rats after 6 weeks of PMA-Zeolite supplementation [7]. In several organs, decreased levels of contaminants were measured, for example, a reduction in Cd in the serum of the small intestine and a reduction in Ni in the kidney [35]. In all these in vivo studies, one common denominator may be observed, which is the fluctuation of metal contaminants and mineral levels during the time-course as observed in humans or in different organs in animals. This may point to the activation of certain metabolic and detoxification processes that would require a more detailed analysis for reaching definite conclusions. Still, major contaminants’ levels were affected and the excretion of certain contaminant metals from the body upon PMA-Zeolite intake was quantifiable and time-dependent. For example, longer periods were required for excretion of Pb than for Al, which is dictated by the metal pharmacokinetics and own-body detoxification mechanisms.

At last, adsorption or binding properties of PMA-Zeolite for D-glucose were recently studied in a model in vitro and in vivo. The study by Markoska et al. [36] has shown that an interaction of D-glucose occurs with the surface of the tested zeolites. Observed D-glucose binding properties were validated in mice pretreated with PMA-Zeolite followed by D-glucose feeding. A 13% decrease in blood glucose levels was observed in mice pretreated with PMA-Zeolite and fed with D-glucose supplementation after 15 min. This specific affinity toward D-glucose should, however, be studied in more detail in vivo due to its potential for hyperglycemia management in humans.

## 3. PMA-Zeolite and Inflammation

One of the most energy-demanding pathological processes in the human body is the inflammatory process, which literally involves the entire organism. A quick reaction of the organism physiology is required to cope with the damage caused by inflammatory processes and restoration of the organism’s disturbed balance. Continuous maintenance of balance in the organism represents an enormous effort in terms of energy and functionality. The functionality, in particular, depends not only on the organism’s ability to manage and restore the entire body system, but also on the efficiency and sensitivity of many receptors and enzymes, as well as the quality of signal transmission to key centers in our brain. For an uninterrupted maintenance of homeostasis in biological systems, a very precise and continuous perception of all external and internal factors with effective receptor and enzymatic systems communication is required. In situations where external or internal stimuli are not correctly perceived, it is necessary to pay attention to the correct and precisely timed impact on the receptors and enzymes and not on the signal responses of the nervous or endocrine systems [37,38]. Unfortunately, no drugs have been developed that efficiently and systemically impact this receptor/enzyme complex system, because drugs are designed to replace the bonds in these biochemical processes that often lead to unwanted side effects. As a consequence, the body’s natural functions, by definition, can no longer act independently. Scientific studies, especially those conducted on in vivo animal models, and some on humans, precisely indicate that that PMA-Zeolite acts systemically. For example, studies on animals have shown that natural zeolite clinoptilolite has systemic immunostimulant effects and is a proven modulator of anti-inflammatory and pro-inflammatory mechanisms [39,40]. The same material from these pioneer studies has been later tested under a new registration name, namely PMA-Zeolite, in inflammation-related human conditions within a clinical study in patients with irritable bowel syndrome (IBS). The study showed a reduction in high sensitivity C-reactive protein (hsCRP) in human blood and a reduction in α1-antitrypsin (α1-antitrypsin) in stool. Both of these parameters are well-established markers of inflammation in the intestines of patients with IBS, and their decreased values are an important indicator of reduced inflammatory environment in the intestine of tested subjects [41]. Similarly, Lamprecht et al. [42] have shown that upon 12 weeks of PMA-Zeolite supplementation in a cohort of aerobically trained subjects, zonulin levels were reduced, which is indicative of improved intestinal wall integrity. In parallel, increased IL-10 levels in the blood were measured as well. IL-10 is an anti-inflammatory cytokine and authors correlate its increased levels with observed anti-inflammatory processes triggered by PMA-Zeolite supplementation.

The effects observed in the above-described studies make it plausible to assume that PMA-Zeolite probably provides support to the body in supplying the receptors/enzymes system with the necessary micro and macro elements both as a result from direct detoxification effect, i.e., removal of harmful contaminants cations in the intestine, as well as due to the release of essential micro elements from its own lattice (i.e., K^+^, Na^+^, Mg^2+^), which then enables the receptors/enzymes’ proper functioning and the proper functioning of each cell. PMA-Zeolite, accordingly, seems to act not selectively, but independently from the ongoing biochemical processes in the cells. This is a highly relevant property supported by in vivo studies of PMA-zeolite effects within the human body. It should indeed be noted that such a conclusion stems from the PMA-Zeolite affinity toward different cations, metals or ammonia, and nitrate ions in the ion-exchange process. Especially, the selectivity alignments given by different authors point, for example, to high affinity toward Ba^2+^, Cd^2+^, Cs^+^ and Pb^2+^ [8]. Zeolite clinoptilolite materials have also a particular affinity toward ammonia cations [43] and this may, accordingly, be assumed among main mechanisms of action of this material in the intestine, especially when the diets are rich in proteins, such as in professional athletes. Moreover, local stimulation of Payer patches, probably as superantigens, which results in diverse mechanisms of immunomodulator molecule production, may be central to observed anti-inflammatory effects. Besides anti-inflammatory cytokines, production of IgA upon zeolite clinoptilolite ingestion should be studied in more detail, as it bears the function of first-line defense on mucous membranes where it prevents access of antigens to the submucosa and circulation [44]. At last, immunodeficiency patients showed increased immune cell counts, HLA-DRC activated T-lymphocytes, CD19C B-lymphocytes and CD4C T-lymphocytes when supplemented with tribomechanically milled zeolite clinoptilolite [45].

## 4. PMA-Zeolite and Osteoporosis

According to promising positive and systemic effects of clinoptilolite both in animals and humans observed in recent decades [8,46,47,48], clinical application of the biologically active substance PMA-Zeolite attracted special attention in the area of bone health, especially due to increased problems with osteoporosis worldwide, partially correlated with increased environmental pollution as well [49,50]. Osteoporosis is a chronic, serious pathology both for the sufferer and for the entire society as a whole because it also represents a demanding public health problem. Clinical studies show that usage of available drugs for a long period of time do not exclude problems in the quality of life, mainly due to increased fracture risk. Some of the current osteoporosis drugs indeed have severe side effects. The first proof of concept study in this field for PMA-Zeolite was performed by Kraljevic Pavelic et al. on animals and humans [51]. Firstly, ovariectomized rats were induced via experimental osteopenia and consequently fed with PMA-Zeolite for 16 weeks. The PMA-Zeolite treated animals had significantly higher bone formation and reduced bone resorption values than after ovariectomy. A clinical intervention with PMA-Zeolite for 1 year was also performed, consequently, in human osteoporotic patients. A significant increase in the bone mineral density, significant pain reduction and significant improvement in quality of life compared with patients in control group was measured at the end of 1 year PMA-Zeolite intervention. This proved a potential effect of PMA-Zeolite on bone integrity in osteoporosis patients. Subsequently, the clinical study was extended for an additional 4 years, which makes a total of 5 years with positive outcomes. The major results show a significantly decreased risk of fractures in PMA-Zeolite-supplemented patients. This may explain the measured levels of bone remodeling markers osteocalcin and beta-cross lap values at the beginning of therapy that all together point to an improved bone strength [52].

**Figure 2:**
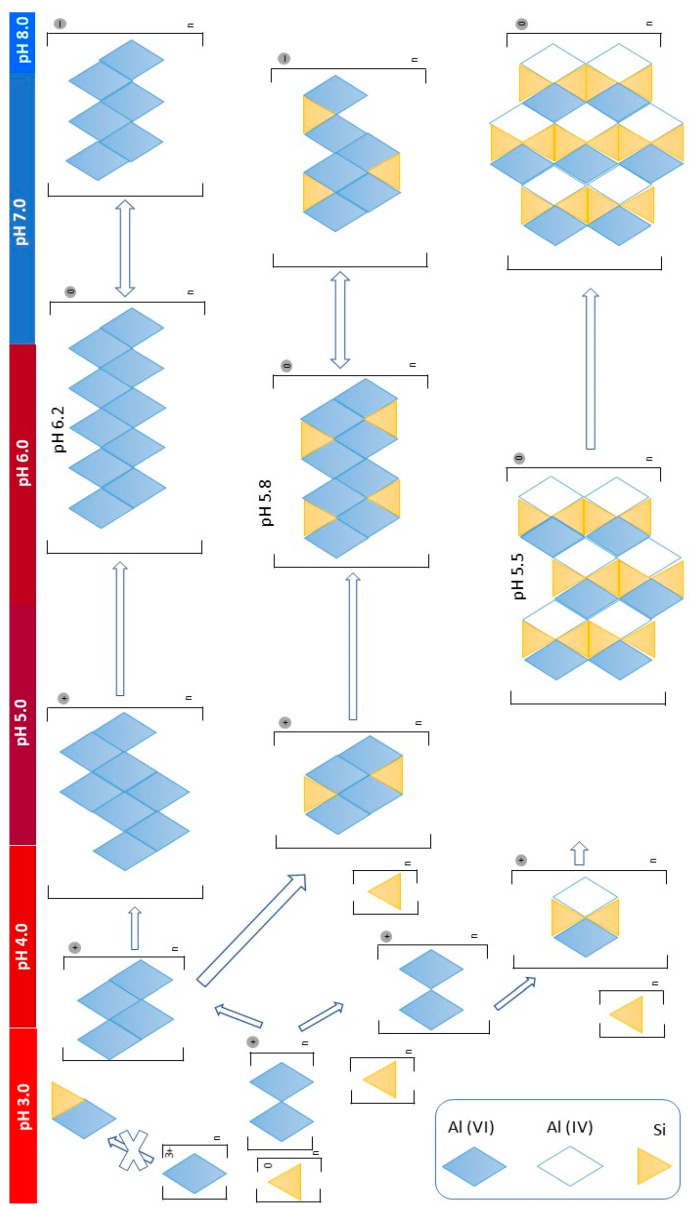
A representation of the pH-dependent formation and stability of Al(OH)_3(_s), hydroxyaluminosilicates HAS with abilities to carry charge, their pH of minimum solubility and their aggregation toward filterable sizes as suggested and explained in the context of reactions with Si(OH)_4_ in more detail by Exley [63]. Figure adapted also from Exley [63].

A certain number of studies conducted on diseases associated with bone mineralization processes, collagen synthesis, pathological changes in the skin and nails, atherosclerosis and Alzheimer’s disease indicate the essential role of silicon in the human body [53,54,55,56]. Specifically, soluble silica forms are relevant to human physiology and especially the orthosilicic acid (H_4_SiO_4_ or Si(OH)_4_), with high bioavailability properties in humans and animals. Important sources of orthosilicic acid are natural mineral waters and natural zeolites, i.e., the zeolite clinoptilolite, colloidal silicic acid and silica gels. Orthosilicic acid may be released from these sources in small, yet significant equilibrium concentrations when the materials come into contact with water or physiological fluids [57,58,59]. Release of essential amounts of orthosilicic acid by PMA-Zeolite as proven in the recent paper by Markoska et al. [36] may underlie part of the observed positive effects of PMA-Zeolite on bones. The therapeutic and biological effects of this phenomenon might be correlated with observed bone formation and bone density in animals with osteopenia supplemented with PMA-Zeolite and consequently on osteoporosis in patients. It could, therefore, be postulated that PMA-Zeolite may exert a positive role in other pathologies where orthosilicic acid may provide benefits, including Alzheimer’s disease, immunodeficiency or skin and hair conditions. A possible mechanism of action might include first the aluminum detoxification properties of soluble silica forms in the body (Figure 2). As aluminum is stored in bones where it has a detrimental effect on the bone architecture and homeostasis [60], a remodeling of the bone tissue may occur due to increased levels of orthosilicic acid, which results in improved bone status. In addition, the positive effects may be envisaged in organs as well, especially in the brain. Recently, a 36-year multicentric study revealed a statistically significant trend for increased aluminum levels in Alzheimer’s disease, dialysis dementia syndrome and Down’s syndrome which clearly correlates aluminum with these pathologies [61]. In addition to aluminum detoxification properties, orthosilicic acid inhibits RANKL-induced osteoclastogenesis, which can also be correlated with improvement of the bone status. In a recent study, authors proved that orthosilicic acid inhibited RANKL-induced osteoclastogenesis and reversed ovariectomy induced bone loss in vivo [62]. Additional scientific research into biological functions of orthosilicic acid would provide more insight into the biological effects and potential therapeutic properties of soluble silica and its sources in humans. However, Si(OH)_4_ and the formation of HAS forms have been found to be protective for humans against biologically available Al exposure (Figure 2).

According to Exley and his pioneer studies [63], the chemistry of mono- or orthosilicic acid (Si(OH)_4_) in the biological context has barely been considered so far. Usually, it is described in processes of autocondensation and reactions with ammonium molybdate. Its unique inorganic chemistry is potentially relevant for the whole biological evolution on Earth; however, it underlies reactions with aluminum (Al) and specifically aluminum hydroxide (Al(OH)_3_(s)) in forming hydroxyaluminosilicates (HAS(s)). HAS_A_ or HAS_B_ can be formed in dependence of the Si:Al ratio upon substitution of Al(OH)_3_ with Si(OH)_4_ (Figure 2). HAS_A_ has octahedrally coordinated Al, Al^VI^. HAS_B_ has equal quantities of Al^VI^ and tetrahedrally coordinated Al, Al^IV^. The formation of HAS_B_ is underlined by Si(OH)_4_-spurred dehydroxylation reaction. HAS_A_ has the ratio of Si:Al 0.5. and its existence is required for the formation of HAS_B_ with the ratio Si:Al is 1.0. In comparison with the amorphous Al(OH)_3_, HAS forms “kinetically” more stable. Exley also proposed that HAS_B_ predominates at pH > 4.0 and [Si(OH)_4_] > 0.1 mmol/L [63].

**Figure 3:**
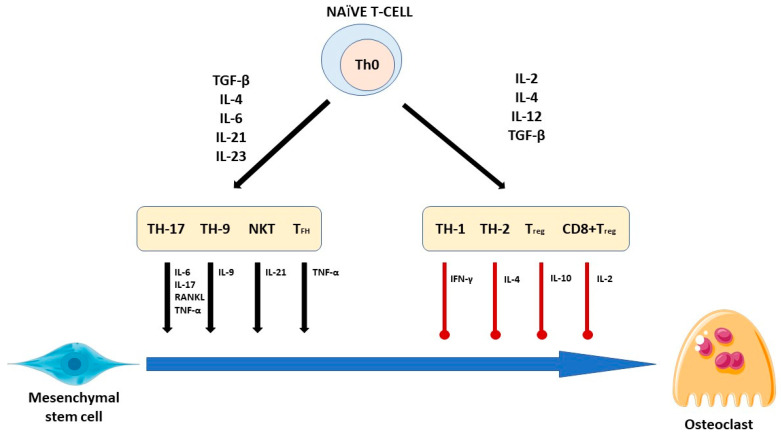
T cells secrete various cytokines that modulate osteoclastogenesis. Naive T cells differentiate according to the secreted pro-inflammatory and anti-inflammatory cytokines conditions, which leads to the development of differentiated T cell subtypes. Mesenchymal stem cells may secrete RANKL which add to other soluble cytokines that promote or inhibit osteoclastogenesis. Adapted from [66].

The second mechanism that may be correlated with positive PMA-Zeolite effects on the bones observed in preclinical and clinical studies may involve local effects of PMA-Zeolite in the intestine, including interactions with the microbiota, where an immunostimulatory effect through Payer’s patches may be deduced as suggested by Kraljevic Pavelic S. et al. [8] and as proven by Lamprecht et al. [42]. Lamprecht et al., indeed, found increased levels of the pro-osteogenic IL-10 upon PMA-Zeolite supplementation in athletes (Figure 3). Both impaired immunity and the microbiome disbalance or dysbiosis have been proven as underlying conditions in osteoporosis development, not excluding the cases associated with the hormonal etiology [64,65,66].

## 5. PMA-Zeolite Applications in Oncology

Research of tribomechanically milled zeolite clinoptilolite in oncology medical applications has been introduced by the group of Prof. Kresimir Pavelic since 2001 [40]. Since then, a number of studies have been conducted that provide evidence on the antiproliferative properties of the zeolite clinoptilolite in tumor cells in vitro [67] and antitumor and antioxidative properties in vivo [39,68]. in vivo results are particularly relevant, and studies on mice and dogs with a variety of tumor types showed a clear decrease in the tumors’ sizes with concomitant positive effects on the overall health status and lifespan [40]. Observed antitumor effects in these studies are probably underlined by a systemic mechanism activated or enhanced by the zeolite clinoptilolite, i.e., detoxification effects, antioxidant effects and immunostimulation. In addition, the zeolite clinoptilolite binds histamine at high affinity and this may also be hypothesized to corroborate positive biological effects in the gastrointestinal tract during the chemo- and radiotherapy of tumors [69,70]. Due to these positive results in the field of oncology, PMA-Zeolite has been recently tested in a clinical set-up as well. Two clinical trials were conducted on oncology patients diagnosed with colon cancer receiving oxaliplatin-containing chemotherapy [71,72]. The oxaliplatin-based treatment is known to cause significant side effects and substantial decrease in life quality of patients. In particular, platin-based drugs, including oxaliplatin, are known to damage peripheral nerves which results in chemotherapy-induced peripheral neuropathy. In the first clinical study, the PMA-Zeolite-treated patient cohort showed better tolerance toward chemotherapy and decreased hematologic toxicity. The male subgroup of patients showed a statistically significant improvement in chemotherapy-induced peripheral neuropathy as well [71]. As these results showed potential, the study was prolonged for an additional 30 months. Positive trends were documented for the overall survival parameter, comorbidities and number of cycles of the oxaliplatin treatment in comparison with the placebo group [72].

**Table 2:** Major observed effects of PMA-zeolite and other clinoptilolite materials in controlled clinical studies.

Clinical Study	Observed Effects	Reference
Randomized, double-blind clinical trial with PMA-zeolite on osteoporosis patients.	- Risk of fractures was significantly decreased in PMA-zeolite-treated patients with respect to time before entering the study (*p* = 0.002). - Osteocalcin and beta-cross laps values show a prevalence of the bone formation process (*p* < 0.05). - BMD values were not significantly affected after the 5-year follow-up in PMA-zeolite-treated patients in comparison with the placebo group.	[7,52]
Blood parameters evaluation in healthy volunteers, Morbus Crohn patients and osteoporosis patients supplemented with PMA-zeolite.	- Levels of aluminum (Al) or Pb were not detected 1 h after intake in the short-term supplementation trial. - Nickel (Ni) and Al were statistically significantly decreased upon long-term 4-year supplementation trials. - Arsenic (As) was statistically significantly decreased upon 12-week supplementation trial. - Alterations were observed for levels of Na and Ca, as well as for Pb, in the long-term trial.	[33]
Prospective, randomized, controlled, double-blind pilot study with PMA supplementation in patients with irritable bowel syndrome (IBS).	- Decreased IBS-associated symptom scores (*p* = 0.001). - Inflammation-related laboratory parameters decreased (reduction in α1-antitrypsin, (*p* = 0.037), lowered inflammation marker hsCRP, microbiome changes (Lactobacillus, Bifidobacteria and Firmicutes).	[41]
Randomized, double-blind placebo-controlled trial with PMA supplementation in trained men and women.	- After 12 weeks, zonulin was significantly decreased (*p* < 0.05) and IL-10 increased tendentially (*p* < 0.1) in the supplemented group.	[42]
Randomized, double-blind, placebo-controlled trial of oral PMA-Zeolite to prevent chemotherapy-induced side effects, in particular, peripheral neuropathy.	- Reduced chemotherapy-induced peripheral neuropathy (CIPN) in men (*p* = 0.047). - Patients treated with the PMA-zeolite were able to undergo more cycles of chemotherapy (*p* = 0.03).	[71]
Follow-up randomized, double-blind, placebo-controlled trial of oral PMA-zeolite to prevent chemotherapy-induced side effects, especially peripheral neuropathy.	- Borderline statistical significance for overall survival for first-line chemotherapy patients at the time point (7 months) (*p* = 0.05) for cancer progression events.	[72]
Non-interventional study in irritable bowel syndrome (IBS) patients supplemented with PMA-zeolite by use of quality of life SF-36 questionnaire, ROM-III criteria improvements of IBS-related symptoms and stool consistency assessment (Bristol Stool Scale).	- Seven of the eight subscales of the SF-36 improved significantly (*p* < 0.001). - Reduction in abdominal pain (*p* < 0.001). - Reduction in abdominal pain and reduction in days with bloating (*p* < 0.001). - The Bristol Stool Scale improvements; (*p* < 0.001).	[73]
Prospective, randomized, open-label, phase I trial with a purified clinoptilolite-tuff (PCT) in artificial wounds in healthy male volunteers compared to the standard of care (SoC).	- No significant differences in wound healing or wound conditions were observed compared with SoC-treated wounds. - Significantly higher proportion of CD68-positive cells. - Significantly lower proportion of α-smooth muscle actin-positive cells in PCT-treated wounds.	[74]
Randomized, placebo-controlled, double-blind pilot study with patients with the clinoptilolite-based product G-PUR^®^ in IBS-D patients.	- After 4 weeks of treatment, 36% of patients in the G-PUR^®^ group vs. 0% in the placebo group reported complete or considerable relief. An improvement in daily abdominal pain was noted in 94% vs. 83% (*p* = 0.0353), and the median number of days with diarrhea per week decreased by 2.4 d vs. 0.3 d in the G-PUR^®^ and placebo groups, respectively.	[75]
A randomized, placebo-controlled, double-blind study to evaluate the effect of G-PUR^®^ on enteral lead isotope ^204^Pb-absorption.	- Concomitant oral intake of purified clinoptilolite-tuff reduced enteral uptake of ^204^Pb in healthy humans by approximately 90%.	[76]
A phase I/IIa prospective, open-label, uncontrolled, dose/granule size-ranging study of a tribomechanically activated clinoptilolite effect on blood lipid profile.	- All lipid fractions significantly improved after 8 weeks of treatment (20–25%, *p* < 0.001), which reversed to baseline after 6 weeks of clinoptilolite withdrawal.	[77]
Randomized clinical trial to evaluate activated and micronized zeolites as modulators of cellular oxidative stress in Mexican smokers without lung diseases.	- Subjects administered activated and micronized zeolites had equivalent antioxidant activities as subjects administered vitamin E.	[78]
Clinical study on healthy volunteers to evaluate the ability of activated clinoptilolite suspended in water to remove heavy metals from the body.	- Participants in both groups had increased concentrations of heavy metals in the urine. - No clinically significant alterations in serum electrolyte levels were observed.	[79]

## 6. Conclusions

Even though natural zeolite clinoptilolite usage in animals and humans has been widely studied over the decades, positive, focused clinical studies have been pursued only recently. Among these studies, a registered zeolite clinoptilolite product PMA-Zeolite has been tested in healthy volunteers, athletes, Morbus Crohn patients, patients diagnosed with osteoporosis, colorectal patients undergoing chemotherapy and patients with irritable bowel syndrome. PMA-Zeolite proved safe in these clinical studies as no side effects were reported in the presented published research. Supplementation of patients or volunteers with PMA-zeolite led to positive systemic health effects; in particular, this was visible in the osteoporosis study where the subjective assessment of overall health conditions at the end of the study after five years of PMA-supplementation was statistically improved [52]. Other positive systemic health effects included lower levels of contaminants (heavy metals) in the blood of healthy volunteers and Morbus Crohn patients, lower levels of markers correlated with oxidative stress and inflammation in colon cancer patients and healthy athletes as well as increased bone strength that led to lower risk of bone fractures in osteoporotic patients. Still, the mechanism underlying these observations on the systemic level of the human body are not fully elucidated. We hypothesize and discuss in the presented paper that observed effects may be correlated with the restoration of biochemical and physiological homeostasis due to (1) the local detoxification properties inside the intestine, i.e., proven removal of heavy metals such as Al, Pb or Cd, (2) superantigen activity on the intestinal mucosa and Payer patches by SiO_2_ components of the material, (3) interactions with the intestinal microbiota, and (4) the release of soluble silica forms, particularly the orthosilicic acid. In particular, release of soluble silica forms from the PMA-zeolite material has been proven in the study by Markoska et al. [36]. According to the literature and herein presented results from the scientific literature, the orthosilicic acid seems to be relevant for removal of Al within the body depos as it enters the bloodstream, which is important for bone health as well. Indeed, Al removal from the bone tissue may be hypothesized to reactivate the bone remodeling process and bone homeostasis. The bone remodeling process upon PMA-zeolite supplementation in the 5-year clinical trial has been proven through monitoring of bone surrogate metabolic markers (bone mineral density, osteocalcin and beta-cross laps) in osteoporosis patients where a clear tendency toward a prevalence of the bone formation process was statistically relevant [57].

The majority of direct PMA-zeolite effects described in this review paper are, however, confined physically to the intestinal lumen and mucosa which are correlated with the nature of the inorganic PMA material. This was witnessed, for example, by a decrease in zonulin values in the blood of tested volunteer athletes as a biomarker for gut permeability [42]. Still, these effects clearly affect the overall human physiology via enhancing the immune status, particularly natural immunity mechanisms through the Peyer patches in the intestine and supporting the body antioxidative mechanisms through local detoxification and positive effects on the intestine in general. Indeed, increased levels of an anti-inflammatory leucine, namely IL-10, was measured in the blood of PMA-supplemented volunteer athletes [42]. These mechanisms were probably relevant also to the observed positive effects in colorectal cancer undergoing oxaliplatin chemotherapy regimens as they lowered the systemic burden caused by chemotherapy, witnessed through reduced chemotherapy-induced peripheral neuropathy in men and an improved completion of the oxaliplatin therapy cycles. Due to positive results in all clinical studies presented herein, and the absence of unwanted side effects caused by PMA-zeolite, the design of larger, multicentric clinical studies may be evaluated to obtain more data on precise clinical applications of PMA-Zeolite and its biological mechanisms of effect.

## Figures and Tables

**Table 1:** Examples of synthetic zeolites applications in vivo. The main types of synthetic zeolites tested for in vivo applications are Metal–Organic Framework (MOF) types of zeolites, mainly Zeolitic Imidazolate Frameworks (ZIFs), Faujasite (FAU) types of zeolites X and Y, Zeolite A/Linde Type A (LTA) zeolites and Zeolite Beta (BEA) types of zeolites.

Material Used in the Study	Zeolite Type	Biological Effect	Reference
Zeolitic imidazolate framework-8-capped ceria nanoparticles (CeO_2_@ZIF-8 NPs)	Zeolitic Imidazolate Framework (ZIF)	- Inhibits lipid peroxidation in brain tissues in middle cerebral artery occlusion mice and reduces oxidative damage and apoptosis of neurons in brain tissue. - Suppresses inflammation and immune response-induced injury. - Neuroprotective action mechanisms of ZIF-8-capped nanomedicine against reperfusion-induced injury in ischemic stroke.	[16]
Zeolitic imidazolate framework 8 (ZIF8)	Zeolitic Imidazolate Framework (ZIF)	- Antibacterial effect on biofilms	[17]
Zeolite imidazolate framework-8	Zeolitic Imidazolate Framework (ZIF)	- Bone regeneration through promotion of angiogenesis, drug loading and antibacterial activity.	[18]
Folic Acid Decorated Zeolitic Imidazolate Framework (ZIF-8)	Zeolitic Imidazolate Framework (ZIF)	- Drug-delivery system (enhanced antibreast cancer effect of baicalin both in vivo and in vitro).	[19]
ZIF-8/gelatin methacryloyl (GelMA) composite hydrogel (GelMA-Z)	Zeolitic Imidazolate Framework (ZIF)	- Reduces the bacterial load, relieves inflammation and promotes alveolar bone regeneration in a rat model.	[20]
Fe_3_O_4_@ZIF-8 nanocomposite carrier was prepared with ZIF-8 as the shell and encapsulated with Fe3O4 by loading DOX to form DOX- Fe_3_O_4_@ZIF-8 (DFZ) drug-loaded magnetic nanoparticles	Zeolitic Imidazolate Framework (ZIF)	- A more substantial antitumorigenic effect as well as superior active tumor targeting of DOX- Fe_3_O_4_@ZIF-8 compared with that of DOX alone.	[21]
Gadolinium-based contrast agents (CAs) based on Faujasite zeolite (NaX) and Zeolite Beta (BEA)	Faujasite (FAU) Zeolite Beta (BEA)	- Improved image contrast.	[22]
Zeolite types X and A, with different Al/Si ratio, ion-exchanged with Ag^+^, Zn^2+^ and Cu^2+^ ions	Zeolite A/Linde Type A (LTA) Faujasite (FAU), zeolite X	- Antimicrobial properties or ion-releasing characteristics to provide prolonged or stronger activity against bacteria, yeast and fungi.	[23]
Zeolitic Imidazolate Framework, Faujasite zeolites and Zeolite A/Linde Type A loaded with anticancer drugs (DOX, 5-FU, curcumin, cisplatin, and miR-34a)	Zeolitic Imidazolate Framework (ZIF), Faujasite (FAU) and Zeolite A/Linde Type A (LTA)	- Enhanced anticancer effect.	[24]

## Data Availability

All data presented in this review paper have been fully cited and are available online.
